# Single-cell and bulk RNA sequencing identifies T cell marker genes score to predict the prognosis of pancreatic ductal adenocarcinoma

**DOI:** 10.1038/s41598-023-30972-7

**Published:** 2023-03-06

**Authors:** Haoran Zheng, Yimeng Li, Yujia Zhao, Aimin Jiang

**Affiliations:** 1 Department of Medical Oncology, Xi’an No.3 Hospital, The Affiliated Hospital of Northwest University, Xi’an, 711018 Shaanxi People’s Republic of China; 2 Department of Medical Oncology, The First Affiliated Hospital of Xi’an Jiaotong University, Xi’an, Shaanxi People’s Republic of China

**Keywords:** Pancreatic cancer, Cancer, Computational biology and bioinformatics, Genetics, Immunology, Biomarkers, Gastroenterology, Oncology

## Abstract

Pancreatic ductal adenocarcinoma (PDAC) is one of the lethal malignancies, with limited biomarkers identified to predict its prognosis and treatment response of immune checkpoint blockade (ICB). This study aimed to explore the predictive ability of T cell marker genes score (TMGS) to predict their overall survival (OS) and treatment response to ICB by integrating single-cell RNA sequencing (scRNA-*seq*) and bulk RNA-*seq* data. Multi-omics data of PDAC were applied in this study. The uniform manifold approximation and projection (UMAP) was utilized for dimensionality reduction and cluster identification. The non-negative matrix factorization (NMF) algorithm was applied to molecular subtypes clustering. The Least Absolute Shrinkage and Selection Operator (LASSO)-Cox regression was adopted for TMGS construction. The prognosis, biological characteristics, mutation profile, and immune function status between different groups were compared. Two molecular subtypes were identified via NMF: proliferative PDAC (C1) and immune PDAC (C2). Distinct prognoses and biological characteristics were observed between them. TMGS was developed based on 10 T cell marker genes (TMGs) through LASSO-Cox regression. TMGS is an independent prognostic factor of OS in PDAC. Enrichment analysis indicated that cell cycle and cell proliferation-related pathways are significantly enriched in the high-TMGS group. Besides, high-TMGS is related to more frequent *KRAS*, *TP53*, and *CDKN2A* germline mutations than the low-TMGS group. Furthermore, high-TMGS is significantly associated with attenuated antitumor immunity and reduced immune cell infiltration compared to the low-TMGS group. However, high TMGS is correlated to higher tumor mutation burden (TMB), a low expression level of inhibitory immune checkpoint molecules, and a low immune dysfunction score, thus having a higher ICB response rate. On the contrary, low TMGS is related to a favorable response rate to chemotherapeutic agents and targeted therapy. By combining scRNA-*seq* and bulk RNA-*seq* data, we identified a novel biomarker, TMGS, which has remarkable performance in predicting the prognosis and guiding the treatment pattern for patients with PDAC.

## Introduction

Pancreatic ductal adenocarcinoma (PDAC) is the most common type of pancreatic cancer, accounting for approximately 90% of cases. Although PDAC is diagnosed in only 13 per 100,000 people annually, its high mortality rate makes it the third leading cause of cancer death in the United States^1–4^. While mortality from other cancers is gradually decreasing, the incidence of PDAC is increasing at a rate of 0.5% to 1.0% per year, and mortality in men is also increasing at a rate of 0.3% per year^1,3–5^. PDAC exhibits an aggressive biological behavior characterized by early metastasis, with a 5-year survival rate of no more than 10%. Only 10–15% of the patients have regional disease suitable for surgery, more than 50% have distant metastases at the time of diagnosis, and the 5-year survival rate of the patients with metastatic disease is less than 3%^1,2,4,5^.

Immunotherapy represented by immune checkpoint blockade (ICB) treatment has revolutionized cancer treatment in the last decade, providing certain benefits to patients with malignancies. However, only approximately 30.0% of patients respond to ICB in most cancer types tested^6^. Up to now, some biomarkers for immunotherapy have been identified, such as tumor mutational burden (TMB), programmed death-ligand 1 (PD-L1), mismatch-repair deficiency (MMR), microsatellite instability (MSI), and gut microbiota^7–9^ However, only a fraction of PDAC patients could benefit from ICB therapy. Clinical trials on cytotoxic T Lymphocyte antigen 4 (CTLA-4) blockade, programmed cell death-1 (PD-1)/ PD-L1 blockade, and dual checkpoint inhibition have poor efficacy, which may be related to the highly immunosuppressive tumor immune microenvironment (TME) of PDAC^5,10–12^. Further exploration of prognostic factors and predictive biomarkers of ICB treatment efficacy in PDAC patients is urgently needed to discover the potential beneficial population of immunotherapy^13–15^.

In recent years, single-cell RNA sequencing (scRNA-*seq*) has been used to study the transcriptomes of different cell types. Compared to traditional RNA-*seq*, it uses optimized next-generation sequencing technology to define the gene expression profile of individual cells, thus helping to dissect the heterogeneity in cell populations^16,17^. Given this advantage, many studies have focused on exploring novel biomarkers of malignancy by integrating scRNA-*seq* and traditional bulk RNA-*seq*^18–21^. Here, this study is designed to identify a robust T cells marker genes score (TMGS) to predict the prognosis and immunotherapy response of PDAC by integrating scRNA-*seq* and traditional bulk RNA-*seq* data.

## Materials and methods

### Raw data acquisition and processing

A total of 11 publicly available datasets from the Gene Expression Omnibus (GEO), The Cancer Genome Atlas (TCGA), and the International Cancer Genome Consortium (ICGC) were used in this study. Nine 10× scRNA-*seq* data of six PDAC samples and three adjacent samples from the GSE212966 series were obtained for scRNA-*seq* analysis. The bulk RNA-*seq* profile of the TCGA-PDAC cohort was employed as a training cohort for model development. Meanwhile, two large PDAC cohorts from the ICGC database and five series from the GEO database were selected as external validation cohorts. Besides, the clinicopathological parameters of each cohort were also downloaded for survival analysis and clinical relevance analysis. The Simple Nucleotide Variation (SNV) data from the TCGA database was also downloaded for TMB calculation and OncoPrint plot generation. All RNA-*seq* data were converted to transcripts per million (TPM) format and further log_2_ transformed. When processing the expression matrix in GEO datasets, each series’ corresponding platform annotation file was downloaded to convert the probes into gene symbols, and the mean expression level of multiple probes that corresponded to the same gene symbol was regarded as the expression level of the corresponding gene. The “voom” function in the “limma” package was applied to raw data normalization. This study also used two immunotherapy (anti-PD-L1/ anti-PD-1) cohorts. Of these, the IMvigor210 cohort contains the complete clinical and expression profile of metastatic urothelial cancer (mUC) patients receiving anti-PD-L1 therapy and was acquired via the R software, “IMvigor210CoreBiologies” package^22^. GSE135222 series records the detailed clinical information and expression matrix of advanced non-small cell lung cancer (adNSCLC) patients treated with anti-PD-L1/ anti-PD-1 agents, which was also obtained from the GEO database for further analysis^23^. The excellent online tool Sangerbox 3.0 was utilized for TCGA and ICGC data acquisition^24^. The detailed information of each cohort used in this study is summarized in Supplementary Table S1.

### scRNA-*seq* data processing and analysis

According to the standardized pipeline, the 10 × scRNA-*seq* data were processed through R software, “Seurat” package. Quality control (QC) was performed on the raw matrix to filter low-quality cells according to the following criteria to obtain a high-quality scRNA-*seq* expression matrix: (1) only genes that were expressed in at least three single cells and cells that expressed more than 250 genes were selected to create a Seurat object; (2) only cells that expressed more than 500 genes and less than 6000 genes were included; (3) the percentage of mitochondrial or ribosomal genes of each cell was calculated and cells that expressed more than 35% of mitochondrial genes were regarded as low-quality cells and were excluded from downstream analysis. Besides, the “LogNormalize” method in the “NormalizeData” function was used to normalize the scRNA-*seq* data and the “FindVariableFeatures” function was adopted to filter the top 2000 highly variable genes after QC. Subsequently, the “RunPCA” function in the “Seurat” package was utilized for principal component analysis (PCA) based on the 2000 genes, and the first 15 PCs were chosen for cell clustering analysis. After that, the “FindNeighbors” and “FindClusters” function in the “Seurat” package was adopted for cell clustering identification, with the parameter “resolution” being set as 0.1. Furthermore, uniform manifold approximation and projection (UMAP)^25^ was used for dimensionality reduction and cluster identification. Then, the “FindAllMarkers” function was exploited to identify significant differentially expressed genes (DEGs) of each cluster by calculating the log_2_ [Foldchange (FC)] and the adjusted *P*-value. DEGs with |log_2_FC|≥ 1 and adjusted *P*-value < 0.05 were considered marker genes of each cluster. The “DotPlot” and “DoHeatmap” function in the “Seurat” package was also adopted to visualize the expression patterns of the top five marker genes in different clusters. Ultimately, R software, “SingleR” package^26^ was employed for automatically cluster annotation to identify the cell types by referring to the Human Primary Cell Atlas. The R software, “UCell” and “irGSEA” packages were used to accomplish single-cell Gene Set Enrichment Analysis (GSEA). The “monocle” package^27^ was adopted for cell trajectory and pseudo-time analysis, with the method “DDRTree” being used for dimensionality reduction. Subsequently, the statistical method “BEAM” was used to calculate the contribution of genes during cell development, and the top 100 genes were selected for visualization. Ultimately, R software, “CellChat”^28^ package was adopted for cell–cell communication network construction. The detailed method was described in the previously published study^29^.

### Subtypes identification based on T cells marker genes

The significant T cell marker genes (TMGs) were mapped into the TCGA-PDAC expression matrix to acquire the corresponding mRNA expression matrix. The Gene Ontology (GO) and Kyoto Encyclopedia of Genes and Genomes (KEGG) enrichment analyses were then performed to explore the biological function of T cells marker genes using R software, “clusterProfiler” package^30^. Subsequently, the unsupervised machine learning algorithm non-negative matrix factorization (NMF) was performed to identify different population subtypes^31^. Briefly, the univariate Cox regression analysis was performed to identify potential prognostic TMGs through “survival” package. Then, R software, “NMF” package was applied for optimal cluster identification based on parameters such as cophenetic, dispersion, and silhouette. GSEA analysis was performed to investigate the biological function difference between the subtypes through the “clusterProfiler” package. Kaplan–Meier survival curves were used to compare their overall survival (OS) and progression-free survival (PFS) difference, with a log-rank test being used for statistical significance determination. We also investigated the association between subtypes and six immune subtypes identified in a previously published study^32^.

### T cell marker genes score construction and validation

The Least Absolute Shrinkage and Selection Operator (LASSO)-Cox regression was performed to screen out the optimal prognostic biomarkers among the 215 TMGs in the TCGA cohort via the R software, “glmnet” package. TMGs with a *P*-value < 0.05 were selected for the LASSO regression analysis to avoid model overfitting, and TMGs with nonzero coefficients were regarded as candidate variables for model construction. In this study, the TCGA-PDAC cohort was designed as a training cohort for model construction, and seven ICGC and GEO cohorts were used as independent external validation sets. Ultimately, the T cells marker genes score (TMGS) was developed based on the regression coefficients (β) derived from the multivariate Cox regression analysis for TMGs with a nonzero coefficient in the LASSO regression. The TMGS calculation formula was developed as below:

$$\mathrm{TMGS}=\sum_{i=1}^{k}\beta i*mRNA\, expression\, level\, of\, (i)$$

The “zscore” function in the R software, “mosaic” package was used for the z-score transformation of TMGS to guarantee their comparability. Patients were classified into the high-TMGS group if the z-score was greater than 0; otherwise, patients were classified into the low-TMGS group. Survival curves were used to visualize the survival difference between high- and low-TMGS groups using R software, “survminer”. The receiver operating characteristic (ROC) curves were used to evaluate the prognostic abilities of TMGS and other single biomarkers, with the area under the curve (AUC) value being calculated to compare their performance using R software, “pROC” package. Besides, time-dependent ROC curves were also used to assess the prediction ability of TMGS in predicting 1-, 2-, and 3-year OS of PDAC via R software, “timeROC” package.

### Clinical relevance and independent prognostic ability of the TMGS

The Wilcox test was performed to investigate the relationship between TMGS and common clinical traits of PDAC in the TCGA cohort. Furthermore, the univariate and multivariate Cox regression analyses were also adopted to evaluate the independent prognostic ability of TMGS compared with clinicopathological characteristics, including age, sex, tumor grade, clinical stage, T stage, N stage, M stage, and alcohol history.

### Biological characteristics, mutation landscape, and immune function status between different TMGS groups

GSEA was performed again to confirm the biological characteristics discrepancy between high- and low-TMGS groups. The “c2.cp.kegg.v7.4.symbols.gmt” gene set was downloaded from the Molecular Signatures Database (MSigDB) for KEGG analysis^33–35^, and the “h.all.v2022.1.Hs.symbols.gmt” gene set was downloaded for hallmark analysis. The R software, “enrichplot” package was used to visualize the top five significantly enriched pathways in GSEA results. The ‘maftools’ R package was utilized to analyze the mutation annotation format (MAF) of different TMGS groups from the TCGA cohort. The waterfall plots were used to summarize their mutation landscape. The single-sample GSEA was implemented to obtain the enrichment level of 29 immune signatures in each PDAC sample via the R software “GSEAbase” and “GSVA” packages. Besides, the Wilcox test was applied to compare each immune function score between high- and low-TMGS groups. ICIs-related genes were also retrieved to investigate their mRNA expression levels between different TMGS groups.

### The role of TMGS in the prediction of immune checkpoint blockade and other therapeutic benefits

The TMB and PD-L1 expression level were widely regarded as predictors of the efficacy of ICB treatment in clinical practice. We calculated the TMB of each patient in the TCGA cohort according to somatic mutation data and compared the TMB value between different TMGS groups. Besides, ROC curves were generated to evaluate the ICB response prediction ability of TMB, PD-L1, and TMGS in PDAC patients. Furthermore, each patient's Tumor Immune Dysfunction and Exclusion (TIDE) score was calculated from the TIDE website (http://tide.dfci.harvard.edu/). The TIDE score was developed to predict anti-PD1 and anti-CTLA4 responses across several melanoma datasets and a limited dataset of NSCLC^36^. In prospective clinical trials, the TIDE score will be of great significance in ICB response prediction^36^.

We also investigated the ICB response predictive ability and risk stratification ability of TMGS in mUC patients (IMvigor210 cohort) and adNSCLC patients (GSE135222 series) who received anti-PD-L1/ anti-PD-1 therapy. The IMvigor210 cohort records RNA-*seq* data, OS, follow-up information, and treatment response information of 348 mUC cases treated with anti-PD-L1 agents. According to patients’ response status, they were stratified into complete response (CR), partial response (PR), stable disease (SD), and progressive disease (PD), respectively. GSE135222 series stores the expression matrix, PFS, survival status, and ICB response of 27 adNSCLC patients. Accordingly, the efficacy was defined as durable clinical benefit [DCB: CR, PR, and SD lasting for ≥ 6 months] or no durable benefit [NDB: PD or SD that lasted < 6 months]^37^. We also applied The R software, “pRRophetic” package to forecast the semi-inhibitory concentration (IC50) values of commonly used chemotherapeutic agents for every PDAC case from different TMGS groups^38^. Ultimately, the Drug Gene Interaction Database (DGIdb) (https://dgidb.genome.wustl.edu/)^39^ was used to find candidate drugs corresponding to the prognostic TMGs, with the PubChem website (https://pubchem.ncbi.nlm.nih.gov/) being exploited to display their 3D structures^40^.

### Statistical analysis

The non-parameter Wilcoxon rank-sum test was used to investigate the relationship of continuous variables between the two groups. Comparison between proportions was evaluated by the chi-square test or Fisher exact test. Kaplan–Meier survival curves were fitted by the “ggsurvplot” function in R software, “survminer” package, and the log-rank test was adopted to examine the statistical difference. The LASSO-Cox regression analyses were applied for TMGS development, with ROC and time-ROC curves being generated for predictive performance assessment^41,42^. A two-sided *P*-value < 0.05 was considered significant. All analyses were conducted in R software (version 4.1.1) for windows 64.0.

## Results

### scRNA-*seq* analysis identifies cell subtypes in PDAC and adjacent tissues

**Figure 1:**
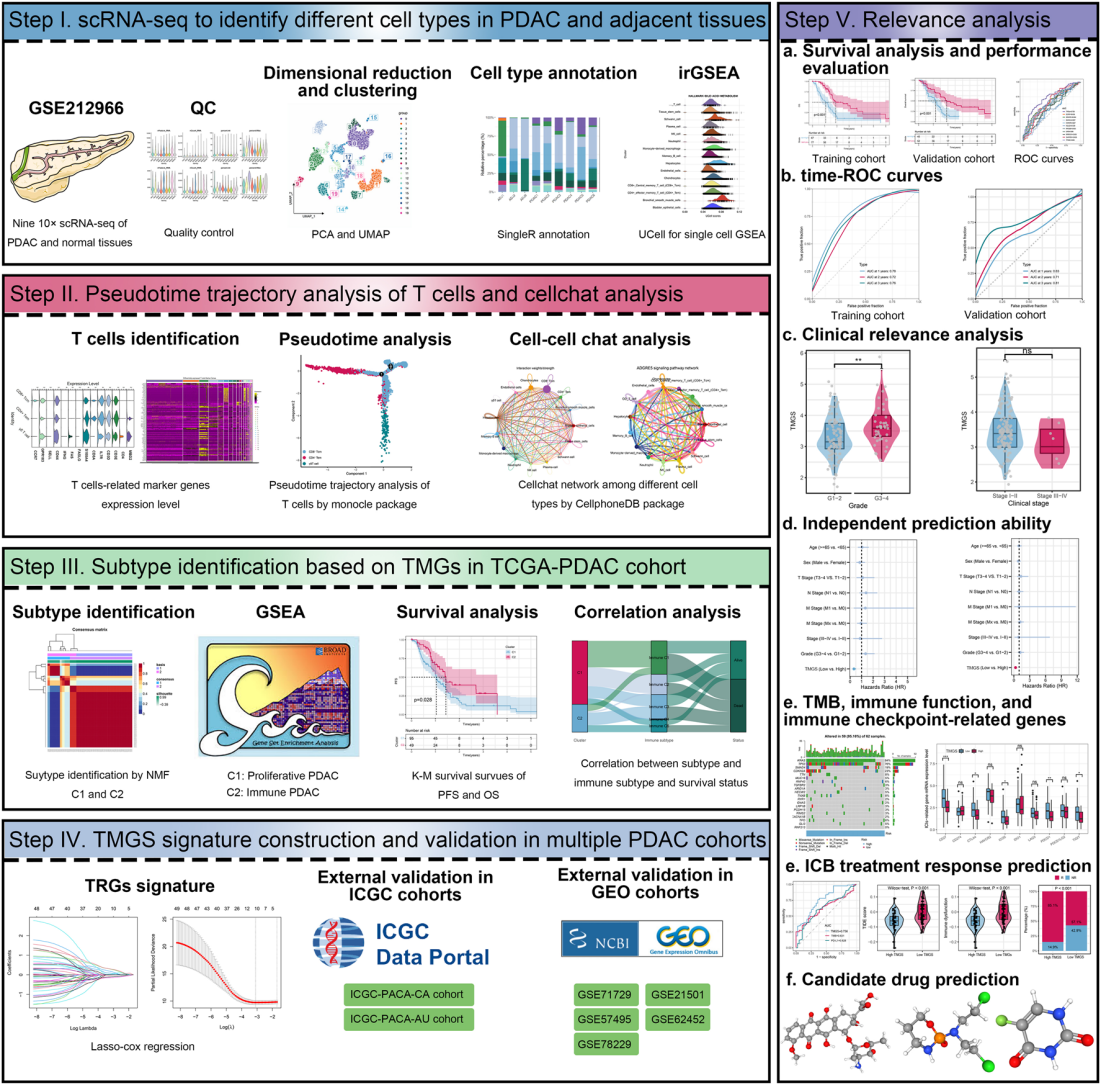
Workflow of the study. scRNA-*seq*, single-cell RNA sequencing; PDAC, pancreatic ductal adenocarcinoma; QC, quality control; GSEA, gene set enrichment analysis; TMGs, T cell marker genes; TCGA; The Cancer Genome Atlas; ICGC, International Cancer Genome Consortium; GEO, Gene Expression Omnibus; ROC, receiver operating characteristics curve; TMB, tumor mutation burden; ICB, immune checkpoint blockade.

**Figure 2:**
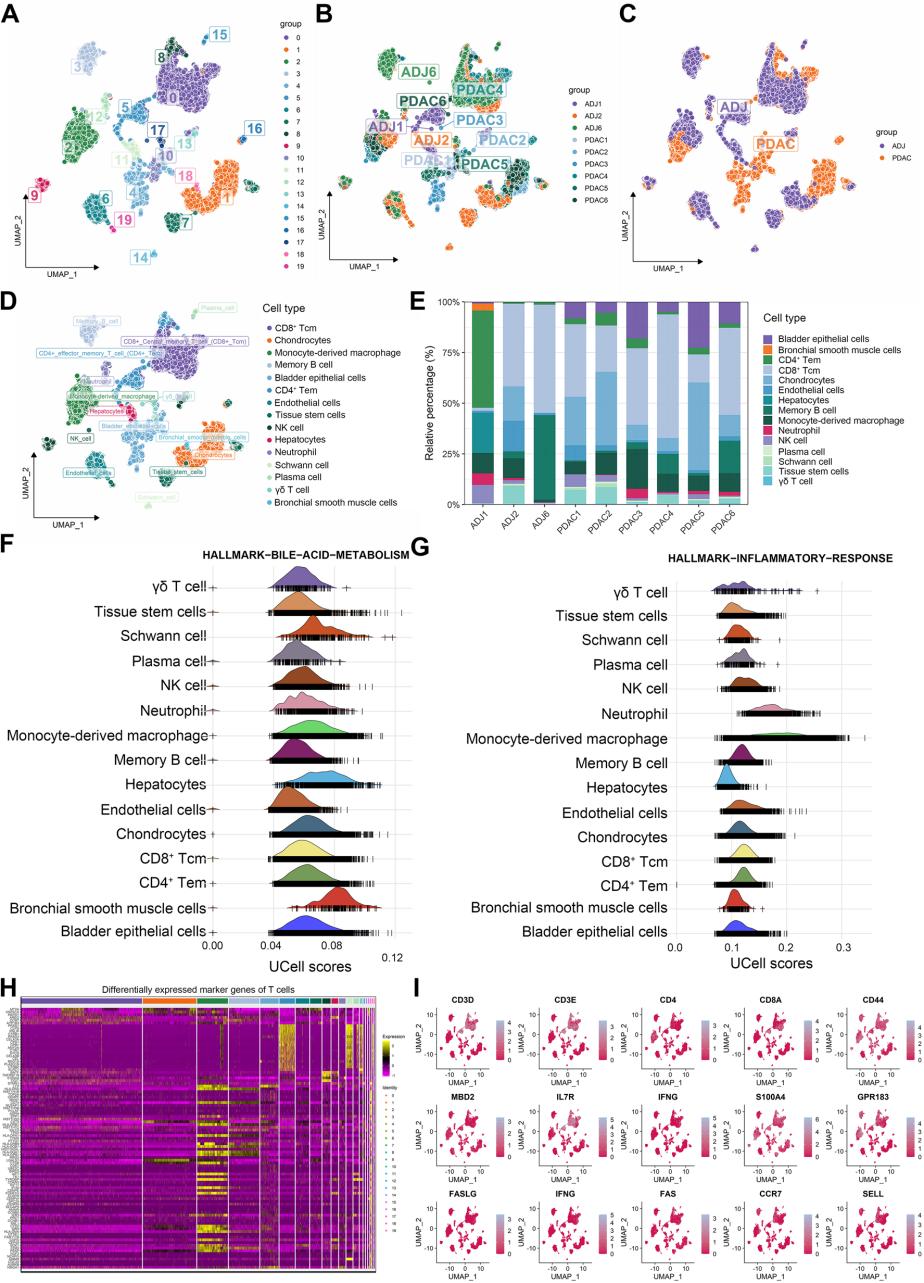
scRNA-*seq* analysis to identify different cell populations in the TME of PDAC and adjacent samples. (**A–D**) UMAP was adopted to identify different clusters (**A–C**) and cell types (**D**) between PDAC and adjacent samples. (**E**) The proportion of different cell types in each sample. (**F–G**) the single-cell GSEA exhibited that all cells were significantly involved in bile acid metabolism (**F**) and inflammation response (**G**) related pathways. (**H**) Heatmap to display the expression pattern of TMGs in different clusters. (**I**) dimensionality reduction plot to show the specific markers genes of CD8^+^ Tcm, CD4^+^ Tem, and γδT cells. scRNA-*seq*, single-cell RNA sequencing; TME, tumor immune environment; PDAC, pancreatic ductal adenocarcinoma; UMAP, uniform manifold approximation and projection; GSEA, gene set enrichment analysis; TMGs, T cell marker genes; TCGA; The Cancer Genome Atlas; TMGS, T cell marker genes score; Tcm, central memory T cell; Tem, effector memory T cell.

The workflow of this study is vividly demonstrated in Fig. 1. A total of 45,029 high-quality cells from six PDAC and three adjacent samples were identified for further analysis after QC (Supplementary Figure S1A, B). Twenty clusters were recognized after running UMAP for dimensionality reduction using 2000 highly-variable genes (Fig. 2A–C, Supplementary Figure S1C, D). The top five marker genes of each cluster were vividly illustrated using the dot plot and heatmap (Supplementary Figure S1E, F). We identified 15 different cell types in these clusters using “singleR” automatic annotation based on the Human Primary Cell Atlas (Fig. 2D). The proportion of different cell types in each sample was displayed in Fig. 2E, depicting that CD8^+^ central memory T cell (CD8^+^ Tcm) is the most predominant cell type in PDAC and adjacent samples (Supplementary Figure S1G). Besides, two other T cells subtype [CD4^+^ effector memory T cell (CD4^+^ Tem) and γδT cell] are identified in these clusters. The single-cell GSEA exhibited that all cells were involved in cell proliferative (such as E2F targets and MYC targets pathways; Supplementary Figure S1H), bile acid metabolism (Fig. 2F), and immune inflammation (Fig. 2G) related pathways.

### T cells expression profile identification and two molecular subtypes determination

**Figure 3:**
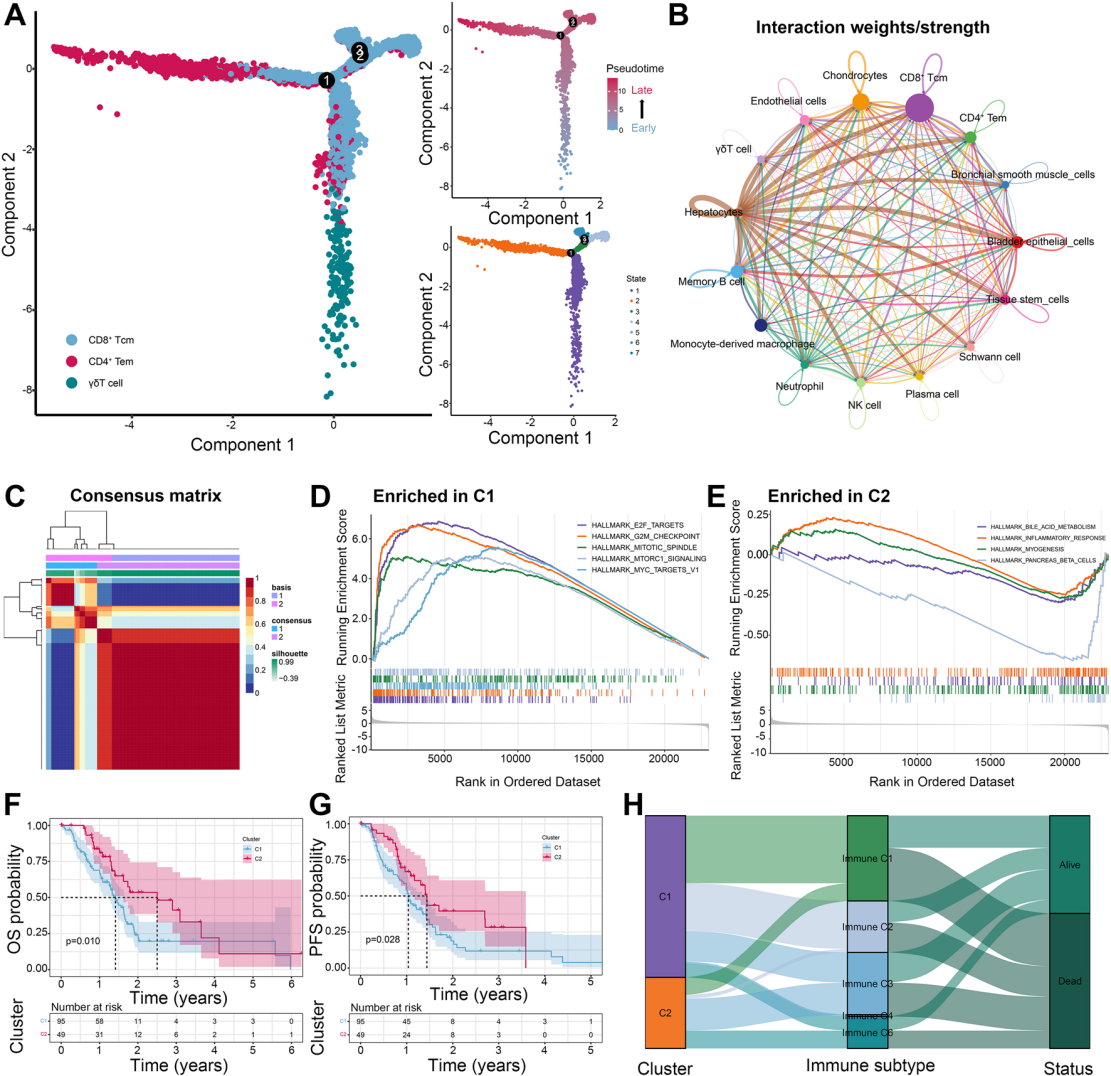
Cell trajectory and pseudo-time analysis of T cells subtypes and identification of two molecular subtypes based on TMGs expression pattern. (**A**) Cell trajectory and pseudo-time analysis of three T cells subtypes. The left panel is the cell trajectory of CD8^+^ Tcm (blue color), CD4^+^ Tem (red color), and γδT cell (green color); The right top panel is the pseudo-time analysis of three subtypes of T cells. The blue color represents the early state of T cells, and the red color represents the late state of T cells; The right bottom panel is the cell fate of three subtypes of T cells. (**B**) Cell–cell communication network between different cell types. The size of the circle and the width of the line represent the interaction weight and strength between them. (**C**) NMF identifies two molecular subtypes in PDAC based on the mRNA expression level of TMGs. (**D-E**) GSEA analysis using gene sets from Hallmark indicated that the C1 subtype was significantly enriched in the E2F target pathway, G2M checkpoint pathway, MTROC1 pathway, and MYC pathways (**D**); C2 subtype was associated with bile acid metabolism, inflammatory response, myogenesis, and pancreas beta cells (**E**). (**F-G**) the OS (**F**) and PFS (**G**) between different molecular subtypes. (**H**) Sankey plot displays the relationship between molecular subtypes, immune subtypes, and survival status. TMGs, T cell marker genes; Tcm, central memory T cell; Tem, effector memory T cell; NMF, non-negative Matrix Factorization; PDAC, pancreatic ductal adenocarcinoma; GSEA, gene set enrichment analysis; OS, overall survival, PFS, progression-free survival.

Given the crucial roles of T cells in cancer immunity and immunotherapy, three T cell subtypes were selected for further analysis. After differential expression analysis, 215 genes were determined as significant T cell marker genes (TMGs). There was a distinct expression pattern of TMGs in different clusters (Fig. 2H). The specific markers genes of CD8^+^ Tcm, CD4^+^ Tem, and γδT cells were displayed in Fig. 2I and Supplementary Figure S1I. Then, cell trajectory and pseudo-time analysis were performed to investigate the dynamic evolution of these three T cell subtypes during PDAC progression. We noticed that γδT cells appeared at the earliest stage of this process, and then CD4^+^ Tem and CD8^+^ Tcm dominated the process (Fig. 3A). Cell–cell communication network indicated that there is crosstalk between T cell subtypes and other cells in the TME of PDAC (Fig. 3B). Furthermore, several pathways were also significantly activated between these cells, including *ADGRE5*, *APP*, *COLLAGEN*, *FN1*, *MIF*, and *SPP1* signaling pathways (Supplementary Figure S2A). GO enrichment analysis illustrated that TMGs were significantly correlated with nuclear division, organelle fission, and mitotic nuclear division (Supplementary Figure S2B). KEGG enrichment analysis indicated that TMGs were associated with pancreatic secretion, cell cycle, protein digestion and absorption, apoptosis, antigen processing and presentation, and p53 signaling pathway (Supplementary Figure S2C). Then, NMF was performed to decipher heterogeneous molecular clusters based on the TMGs expression matrix in the TCGA cohort. Ultimately, all patients were stratified into two molecular subtypes (Fig. 3C), as illustrated in Supplementary Figure S3A. We further conducted GSEA analysis using gene sets from KEGG and Hallmark to understand the biological characteristics discrepancy between the two subtypes. Excitingly, C1 was significantly enriched in the E2F target pathway, G2M checkpoint pathway, MTROC1 pathway, cell cycle, and DNA replication (Fig. 3D and Supplementary Figure S3B), indicating a proliferative phenotype. On the contrary, C2 was associated with bile acid metabolism, inflammatory response, complement and coagulation cascades, and intestinal immune network for IgA production (Fig. 3E and Supplementary Figure S3C), representing an immune phenotype. Therefore, we defined the C1 subtype as proliferative PDAC and the C2 subtype as immune PDAC, respectively. Besides, survival analysis was performed to evaluate the survival difference between these subtypes. The results revealed that C1 was correlated with poor OS (Fig. 3F) and PFS (Fig. 3G) compared with the C2 subtype. Furthermore, we also investigated the relationship between different immune subtypes and molecule subtypes via the Sankey plot. It showed that C1 was mainly classified into Immune C1 (wound healing) and Immune C2 (IFN-gamma dominant) subtypes (Fig. 3H), while C2 was mainly classified into Immune C3 (inflammatory) subtype (Fig. 3H). Therefore, PDAC patients could be classified into two distinct molecular subtypes based on TMGs, with different biological behavior, clinical outcome, and immune landscape.

### TMGS development and validation in multiple PDAC cohorts

**Figure 4:**
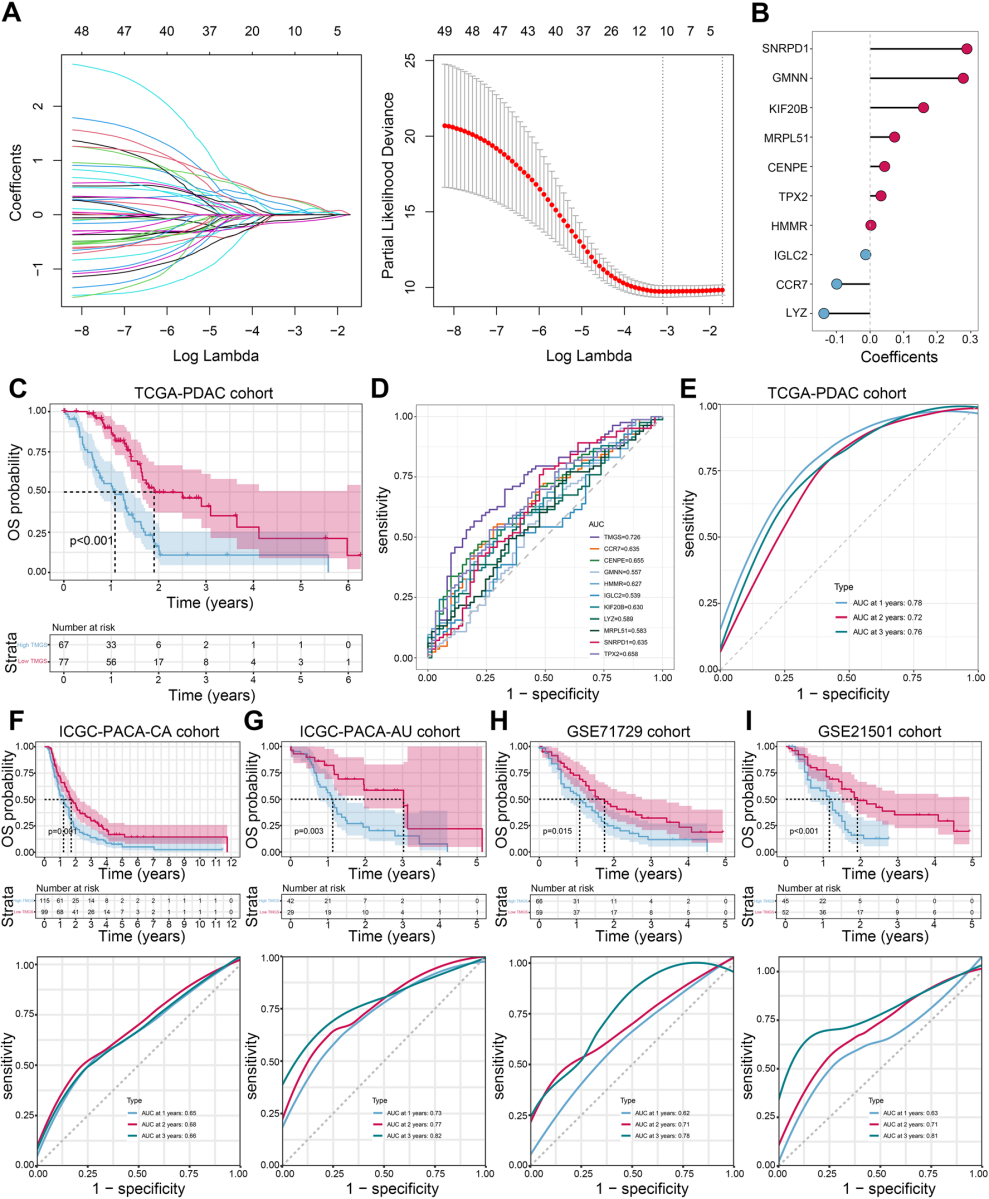
TMGS identification and validation in multi-omics datasets. (**A**) LASSO regression identifies 10 optimal TMGs to develop TMGS. The left panel represents the variable selection process during LASSO regression. The horizontal axis is the penalized parameter lambda after log transformation. The vertical axis is the coefficients of each variable. The coefficients gradually tended to zero with the increment of lambda. Eventually, variables with nonzero coefficients were selected for further analysis. The right panel is the tenfold CV of the LASSO model. (**B**) Dot plot to display the coefficients of the LASSO-identified 10 TMGs. Blue color represents coefficients less than 0, while red color represents coefficients greater than 0. (**C**) Kaplan–Meier survival curve with log-rank test indicated that high TMGS is correlated with poor OS in PDAC. (**D**) ROC curves of TMGS and each TMGs to compare their performance in predicting the clinical outcome of PDAC. (**E**) time-dependent ROC curves to evaluate the performance of TMGS in predicting the 1-, 2-, and 3-year OS probability of PDAC. (**F–I**) External validation of the risk stratification ability and 1-, 2-, and 3-year OS probability prediction ability of the TMGS in ICGC-PACA-CA (**F**), ICGC-PACA-AU (**G**), GSE71729 (H), and GSE21501 (**I**) cohorts. TMGs, T cell marker genes; LASSO, Least Absolute Shrinkage and Selection Operator; CV, cross-validation; TMGS, T cell marker genes score; ROC, receiver operating characteristic curve; OS, overall survival, PDAC, pancreatic ductal adenocarcinoma; ICGC, International Cancer Genome Consortium.

First, the single-factor Cox regression analysis was performed to identify potential prognostic genes in 215 TMGs from the TCGA cohort. A total of 50 TMGS with a *P*-value less than 0.05 were selected for LASSO-Cox regression. After taking variables into LASSO-Cox regression analysis with minimized lambda, ten genes with nonzero coefficients were identified to construct TMGS (Fig. 4A). As depicted in Fig. 4B, the TMGS was calculated according to the following equation: TMGS = -0.148 * *CCR7* + (−0.081) * *CENPE* + 0.452 * *GMNN* + 0.001 * *HMMR* + (−0.03) * *IGLC2* + 0.369 * *KIF20B* + (−0.179) * *LYZ* + 0.174 * *MRPL51* + 0.434 * *SNRPD1* + (−0.007) * *TPX2*. All patients were classified into high- and low-TMGS groups after standardizing the TMGS by z-score. Survival analysis indicated that the high-TMGS group correlated significantly with poor OS than the low-TMGS group (Fig. 4C). ROC curves demonstrated that TMGS has a greater AUC value than other single biomarkers in predicting the clinical outcome of PDAC (Fig. 4D). Meanwhile, time-dependent ROC curves also revealed that TMGS has acceptable performance in predicting the 1-, 2-, and 3-year OS of PDAC (Fig. 4E). Seven independent external validation cohorts were used to verify the risk stratification and predictive ability of TMGS, with consistent results observed in them (Fig. 4F-I and Supplementary Figure S3D). Taken together, TMGS could be a reliable prognostic biomarker for PDAC.

### The TMGS can serve as an independent prognostic factor for PDAC

**Figure 5:**
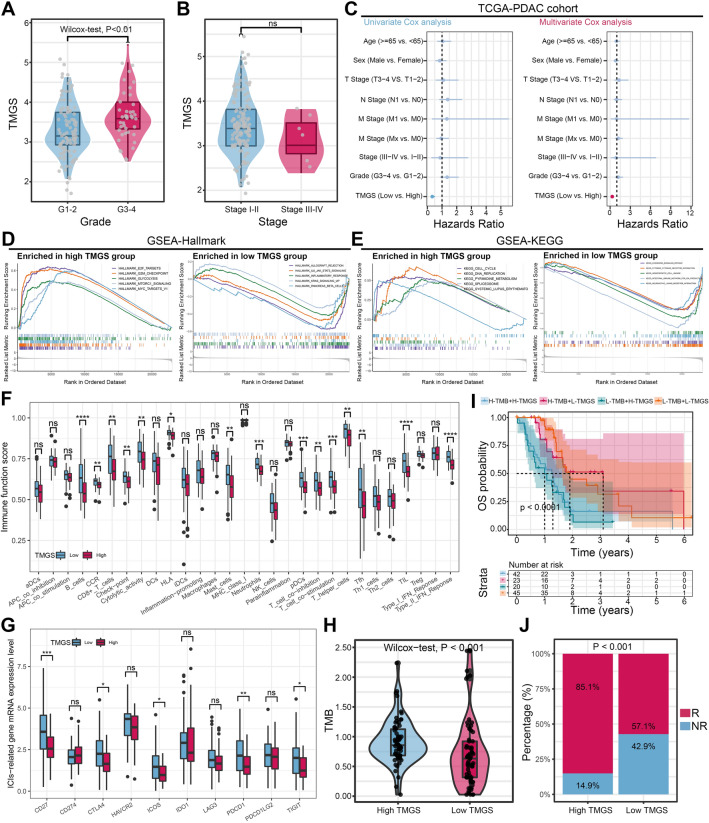
TMGS is an independent prognostic factor of PDAC and is correlated with the response to ICB treatment. (**A, B**) The relationship between TMGS and tumor grade (**A**) and stage (**B**). (**C**) Forest plots to show the independent prognostic ability of TMGS. The left panel represents the result of the univariate Cox regression analysis. The right panel represents the result of the multivariate Cox regression analysis. (**D**) GSEA using gene sets from Hallmark revealed that high-TMGS was significantly enriched in E2F targets, G2M checkpoint, MTORC1 signaling, MYC, and glycolysis pathways. Low-TMGS was enriched in IL6-JAK-STAT3 signaling, inflammatory response, and pancreas beta cell pathways. (**E**) GSEA using gene sets from KEGG revealed that high-TMGS was significantly enriched in cell cycle, DNA replication, and spliceosome pathways. Low-TMGS was enriched in the chemokine signaling pathway, cytokine-cytokine receptor interaction, and IgA production pathways. (**F**) The relationship between TMGS and immune cell infiltration score and immune function score. (**G**) The relationship between TMGS and the mRNA expression level of immune checkpoint molecules (**H**) The relationship between TMGS and TMB value. (**I**) The Kaplan–Meier survival curve with the log-rank test shows the survival difference between different TMGS and TMB groups. (**J**) Bar plot to show the difference in response to ICB treatment between different TMGS groups according to the TIDE algorithm. TMGS, T cell marker genes score; PDAC, pancreatic ductal adenocarcinoma; ICB, immune checkpoint blockade; GSEA, Gene Set Enrichment Analysis; KEGG, Kyoto Encyclopedia of Genes and Genomes; ssGSEA, single sample GSEA; GSVA, Gene Set Variation Analysis; TMB, tumor mutation burden; TIDE, Tumor Immune Dysfunction and Exclusion.

We observed that TMGS was significantly correlated with the clinical outcome of PDAC. Here, we further investigated the relationship between TMGS and common clinical features of PDAC. Not surprisingly, clinical relevance analysis indicated that higher TMGS was significantly correlated with higher tumor grade (G3-4: poor differentiation and undifferentiation, Fig. 5A). However, there was no statistical significance in the tumor stage (Fig. 5B) and other clinicopathological parameters (Supplementary Figure S3E). Then, we evaluated the independent predictive ability of TMGS compared with these clinical traits in the TCGA cohort and external validation cohorts using the univariate and multivariate Cox regression analyses. The results indicated that TMGS is an independent prognostic factor of PDAC in the TCGA cohort (Fig. 5C) and most of the external validation cohorts (Supplementary Figure S4A–F). Hence, TMGS is closely related to the tumor grade of PDAC and can serve as an independent prognostic factor for these individuals.

### Biological characteristics, mutation landscape, and immune function between different TMGS subtypes

We used KEGG and Hallmark as reference gene sets for GSEA enrichment analysis to explore the biological characteristics of different TMGS groups to provide more insight into their different clinical prognosis. Enrichment analysis illustrated that high-TMGS was significantly enriched in processes or pathways associated with cell cycle and cell proliferation (such as E2F targets, G2M checkpoint, MTORC1 signaling, cell cycle, and DNA replication, and glycolysis; Fig. 5D, E). On the contrary, low-TMGS was enriched in immune-related pathways or processes (such as IL6-JAK-STAT3 signaling, inflammatory response, chemokine signaling pathway, cytokine-cytokine receptor interaction, and IgA production; Fig. 5D, E). Afterward, two waterfall plots were generated to explore the detailed gene mutation landscape between high- and low-TMGS groups. We observed that patients in the high-TMGS group harbored more frequent gene alteration than those in the low-TMGS group (95.16% vs. 79.4%, Supplementary Figure S4G). *KRAS*, *TP53*, *SMAD4*, and *CDKN2A* were the most frequently altered genes in both groups (Supplementary Figure S4G). We estimated the 29 immune scores to evaluate the immune function between high- and low-TMGS groups using ssGSEA and GSVA algorithms. It showed that low-TMGS was significantly correlated with more positive immune cells infiltration status, including B cells, CD8^+^ T cells, Mast cells, neutrophils, T helper cells, follicular helper T cell (Tfh), and tumor-infiltrating lymphocyte (TIL) (Fig. 5F). Besides, there was a more active type II IFN response score in the low-TMGS group than in the high-TMGS group (Fig. 5F). Therefore, these results suggested that high-TMGS was associated with proliferative phenotype, more frequent tumor suppressor gene mutation, and unfavorable immune function status of PDAC.

### TMGS-based treatment strategy for PDAC

**Figure 6:**
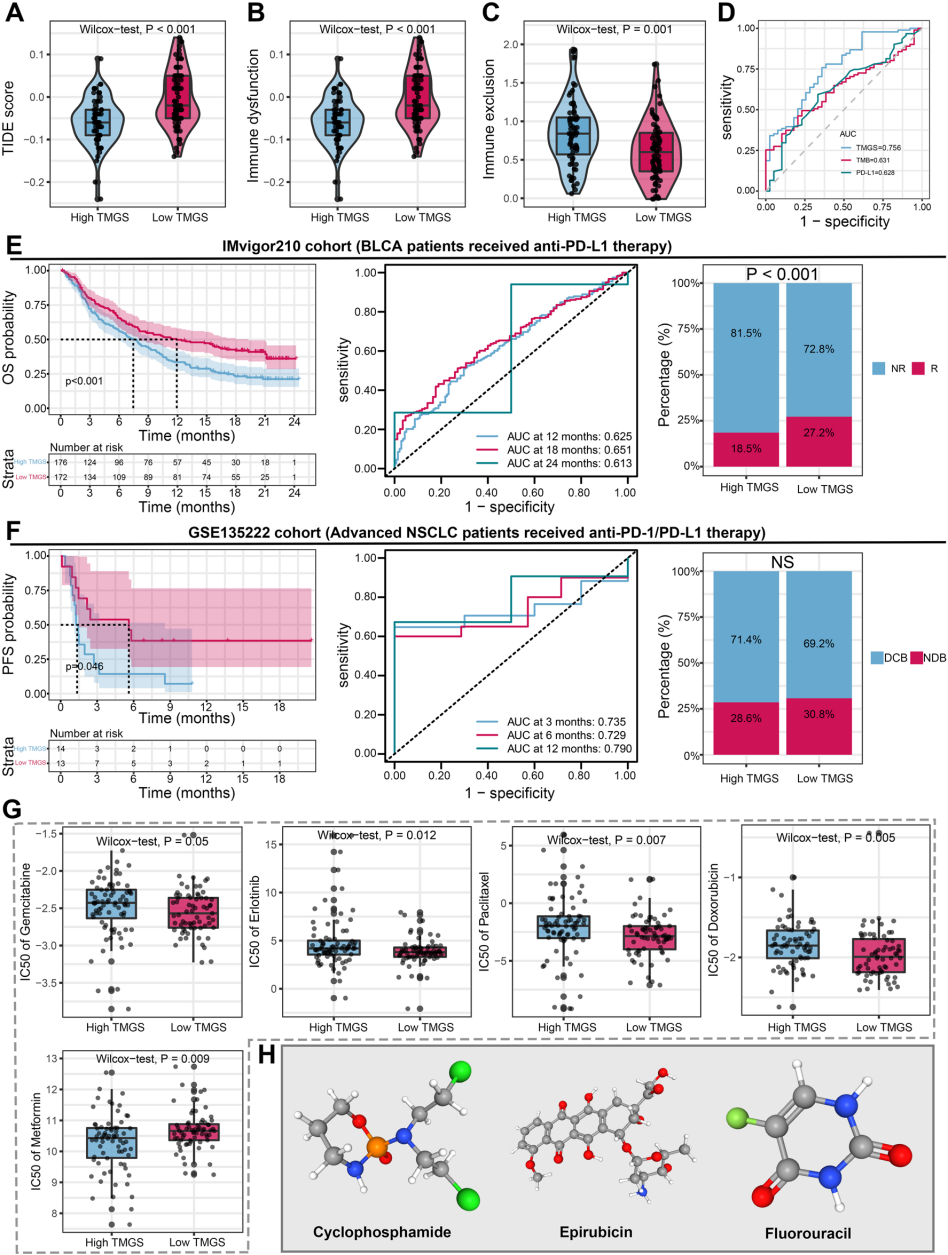
TMGS could guide the treatment patterns of patients with PDAC. (**A–C**) The relationship between TMGS and TIDE score (**A**), immune dysfunction score (**B**), and immune exclusion score (**C**). (**D**) ROC curves to show the performance of TMB, PD-L1 expression level, and TMGS in predicting the response of ICB treatment in PDAC. (**E**) The risk stratification and treatment response predictive abilities of TMGS in the IMvigor cohort. The IMvigor210 cohort records RNA-*seq* data, OS, and treatment response information of 348 mUC cases treated with anti-PD-L1 agents. According to patients’ response status, they were stratified into CR, PR, SD, and PD, respectively. (**F**) The risk stratification and treatment response predictive abilities of TMGS in the GSE135222 cohort. GSE135222 series stores the expression matrix, PFS, survival status, and ICB response of 27 adNSCLC patients. Accordingly, the efficacy was defined as DCB (CR, PR, and SD lasting for ≥ 6 months) or NDB (PD or SD that lasted < 6 months). (**G**) Box plots to display the relationship between TMGS and the IC50 values of Gemcitabine, Erlotinib, Paclitaxel, Doxorubicin, and Metformin. (**H**) The 3D structure tomographs of the three-candidate small-molecule drugs for PDAC. TMGS, T cell marker genes score; PDAC, pancreatic ductal adenocarcinoma; TIDE, Tumor Immune Dysfunction and Exclusion; ROC, receiver operating characteristics curve; TMB, tumor mutation burden; ICB, immune checkpoint blockade; OS, overall survival; mUC, metastatic urothelial cancer; CR, complete response; PR, partial response; SD, stable disease; PD, progressive disease; adNSCLC, advanced non-small cell lung cancer; PFS, progression-free survival; DCB, durable clinical benefit; NDB, no durable benefit; IC50, semi-inhibitory concentration.

The mRNA expression level of immune checkpoint molecules was compared between different TMGS groups. To our surprise, the mRNA expression level of inhibitory immune checkpoint molecules such as *CTLA4*, *ICOS*, *PDCD1* (*PD-1*), and *TIGIT* is significantly upregulated in the low-TMGS group (Fig. 5G). There was no statistical difference in *CD274* (*PD-L1*), *HAVCR2*, *IDO1*, *LAG3*, and *PDCD1LG2* (*PD-L2*) expression levels between the two groups (Fig. 5G). Subsequently, we compared the distribution of TMB and TIDE scores in different TMGS groups to investigate the predictive ability of TMGS to ICB treatment. The results indicated that the TMB was significantly higher in the high-TMGS group than in the low-TMGS group (Fig. 5H). We also performed a subgroup survival analysis to investigate the risk stratification ability of TMGS when combined with TMB level (TMB is divided into high- and low-TMB groups according to its median value). It showed that TMGS still has good risk stratification ability when incorporated with TMB (Fig. 5I). The TIDE score is a widely used biomarker for predicting the response rate to ICB treatment. A lower TIDE score was associated with more favorable ICB therapy efficacy. According to the TIDE algorithm, patients in the high-TMGS group have lower TIDE scores and are prone to have a remarkable response rate to ICB therapy than them in the low-TMGS group (85.1% vs. 57.1%, *P* < 0.001, Figs. 5J and 6A). Besides, we found that high TMGS correlated with a lower immune dysfunction score (Fig. 6B) but a higher immune exclusion score (Fig. 6C). As we all know, TMB and PD-L1 are the most common biomarkers for predicting ICB treatment efficacy in clinical practice. Here, we also compared the performance of TMGS, TMG, and PD-L1 in predicting the response to ICB treatment. ROC curves illustrated that TMGS has a remarkable predictive performance (AUC = 0.756) than TMB (AUC = 0.631) and PD-L1 (AUC = 0.628), suggesting that it could be a candidate efficacy predictive biomarker for PDAC patients treated with ICB (Fig. 6D).

Owing to the relatively few datasets in public databases that contain the PDAC immunotherapy cohort, the IMvigor210 cohort and GSE135222 series were used as test sets to evaluate the potential of TMGS as a prognostic marker and a predictor of ICB treatment response. Survival and time-dependent ROC curves showed that TMGS has good risk stratification ability and prognosis prediction performance in both cohorts (Fig. 6E, F). However, inconsistent with the TCGA cohort, the result in the IMvigor210 cohort showed that patients in the low-TMGS group possess a higher response rate to ICB treatment than those in the high-TMGS group (27.2% vs. 18.5%, *P* < 0.001, Fig. 6E). Besides, there was no statistical difference between the DCB and NCB rates in adNSCLC patients treated with anti-PD-L1 therapy (Fig. 6F). Next, we estimated the IC50 values of commonly used chemotherapeutic drugs for each PDAC patient. By comparing the IC50 values of different TMGS groups, we identified that patients in the high-TMGS group might respond well to Metformin (Fig. 6G). However, the IC50 values of Gemcitabine, Erlotinib, Paclitaxel, and Doxorubicin in the high-TMGS group are significantly greater than those in the low-TMGS group (Fig. 6G). Ultimately, the DGIdb database was utilized to identify potential small-molecule therapeutic drugs for PDAC. The 3D structure tomography of Cyclophosphamide, Epirubicin, and Fluorouracil was found in the PubChem database (Fig. 6H). Hence, TMGS could serve as a valid biomarker to predict the efficacy of ICB therapy in PDAC. Higher TMGS was associated with a more remarkable treatment response to ICB. Besides, patients in the high-TMGS group may have a survival benefit from metformin treatment.

## Discussion

PDAC is one of the lethal malignancies, with limited biomarkers identified to predict its prognosis and treatment response of ICB. With the rapid development of RNA-*seq*, a growing number of studies are conducted to identify the candidate biomarker of PDAC through multi-omics technology^43–50^. This study aimed to explore the predictive ability of TMGS to predict their OS and treatment response to ICB by integrating scRNA-*seq* and bulk RNA-seq data. In this study, we identified and verified a novel biomarker, TMGS, that could effectively predict the prognosis and immunotherapy response of PDAC by combining scRNA-*seq* and multiple RNA-*seq* datasets. We characterized the components of TME in PDAC in a public dataset that contains nine 10 × scRNA-*seq*. We identified three T cell subtypes after cell annotation, including CD8^+^ Tcm, CD4^+^ Tem, and γδT cells. Memory T cells possess the property of long-term remembrance of priming antigens or pathogens and evoke a rapid recall response with enhanced magnitude upon antigen reencounter^51^. According to the expression pattern of specific markers, human memory T cells could be subdivided into Tcm and Tem, with different functional properties observed^51,52^. However, the role of memory T cells in cancer is not well characterized. Recently, Ning and colleagues identified that CD4^+^ memory T cell-based gene risk score is associated with the prognosis of patients with gastric cancer ^53^. Although γδT cells only account for a small part of T cells, they are considered to bridge the innate and adaptive immune systems^54,55^. Recent studies indicated that activated γδT cells could release granzyme B and perforin to trigger anticancer immunity, thereby counteracting tumor development^54,56^. As such, many γδT cells-based approaches are developed to achieve cancer immunotherapy and are currently evaluating the efficacy and safety in clinical trials^54^. Therefore, we performed the current study to develop a novel prognostic biomarker based on the marker genes of three T cell subtypes to predict the prognosis of PDAC in the TCGA cohort. Besides, the performance of TMGS was also verified in seven external PDAC cohorts and two ICB treatment cohorts, indicating that it could serve as an independent prognostic factor. Most importantly, we also found that TMGS has the potential to predict the ICB therapy response in PDAC and other malignancies.

Using an unsupervised learning algorithm, we defined two molecular subtypes (C1: proliferative PDAC; C2: immune PDAC, respectively) in PDAC based on the expression patterns of TMGs in the TCGA cohort. Multiple cell cycle and cell proliferation-related pathways and signaling are aberrantly activated in proliferative PDAC, resulting in bleaker OS and PFS in these individuals. On the contrary, we observed that the immune subtype was related to inflammatory response and immune-related pathways. Thus, a more satisfactory prognosis was achieved in these patients. Then, we calculated TMGS to predict the prognosis of PDAC through a combination of the regularized approach LASSO regression and traditional Cox regression analysis. All patients in the TCGA cohort were classified into high- and low-TMGS groups according to standardized TMGS. There was a distinct clinical outcome between different TMGS groups—high TMGS was related to poor OS, while low TMGS was associated with more favorable OS. The following investigations were conducted to gain insights into the reasons for this survival disparity. First, clinical relevance analysis indicated that there were more G3-4 patients in the high-TMGS group, which explains the lethal prognosis in this cluster. Second, GSEA and GSVA were utilized to explain the cause of this difference in the perspective of biological characteristics. Surprisingly, GSEA analysis demonstrated that many cell cycle and cell proliferation-related pathways were significantly altered in the high-TMGS group, which entitled a more malignant biological behavior of the tumors in this group. On the contrary, we observed that immune and inflammatory response-related pathways were predominantly enriched in the low-TMGS group. Therefore, 29 immune scores were further estimated to evaluate the immune function between high- and low-TMGS groups using ssGSEA and GSVA algorithms. The results showed that the immune function score of type II IFN response is significantly lower in the high-TMGS group, indicating impaired antitumor immunity^57^. Besides, the multiple immune cells infiltration score also indicated an attenuated antitumor immunity in this group, supported by the lower B cells, CD8^+^ T cells, and TIL score. Hence, the impaired anticancer immunity in the high-TMGS group could explain its unideal prognosis. Third, the mutation landscape between different TMGS groups illustrated that the high-TMGS group harbored more *KRAS*, *TP53*, and *CDKN2A* mutation than the low-TMGS group, despite the mutation frequency of *SMAD4* being similar in both groups. The aberrant genetic events in PDAC are generally divided into oncogene activation and tumor suppressor inactivation, and the above four genetic mutations were the most predominant gene alteration in PDAC^58^. *KRAS* mutations are the most prevalent mutations identified in human solid tumors, and approximately 90% of patients with PDAC harbor the G12 mutation in *KRAS*^58^. The most frequent point mutations at G12, G13 and Q61 inhibit the intrinsic GTPase activity of *RAS*, thus sustaining the GTP-bound state of the *RAS* protein, which is established to be oncogenic^58^. As one of the most famous tumor suppressors, *p53* plays a pivotal role in maintaining genetic stability and inhibiting tumor vascularity^58^. *TP53* is the most commonly inactivated tumor suppressor in PDAC^58^. Approximately 70% of patients with PDAC harbor alterations in the *TP53* gene^58^. *CDKN2A*, also known as cyclin-dependent kinase inhibitor 2A, encodes p16 and p19 to arrest the cell cycle at the G1/S checkpoint through a CKD4/6-regulated mechanism^58,59^. Approximately 60% of patients with PDAC harbor *CDKN2A* mutations, and germline mutations in *CDKN2A* are associated with a high risk of developing PDAC^58,60^. Recently, McIntyre and colleagues identified that genomic alterations in *KRAS* and *TP53* are associated with worse OS in patients with resected PDAC^61^. Besides, Qian et al. indicated that *KRAS*, *TP53*, and *CDKN2A* alterations are associated with decreased recurrence-free survival (RFS) and that *CDKN2A* was associated with worse OS in resected PDAC^62^. Therefore, the higher mutation frequency of *KRAS*, *TP53*, and *CDKN2A* in the high-TMGS group also supports its poor prognosis.

Nowadays, cancer immunotherapy represented by ICB has revolutionary changed the treatment patterns of malignancies. However, only a fraction of patients presented durable clinical benefits to this treatment^6^. Lacking reliable predictive markers is one of the most important reasons. Various factors are associated with the response of ICB treatment, including TMB, PD-L1 level, MMR, cytotoxic T lymphocyte infiltration, and gut microbiota^6^. However, none of these factors is sufficient to achieve accurate outcome prediction. Furthermore, identifying ICIs treatment response biomarkers and resistance regulators is a critical challenge in the field^6,63^. Thus, Jiang and colleagues developed the TIDE signature to fill this gap and showed the good predictive ability of ICB therapy^6^. According to the TIDE signature, this study identified that high TMGS is associated with the poor OS but with favorable ICB response. Before this, we identified that the low-TMGS group had higher infiltration of TILs and activated antitumor immunity compared to the high-TMGS group. However, to our surprise, patients in the low-TMGS group have higher expression levels of inhibitory immune checkpoint molecules, including *CTLA-4*, *PD-1*, *ICOS*, and *TIGIT*. This suggests that although there is more infiltration level of TILs in the low-TMGS group, they are switched to exhausted T cells and lose robust antitumor immunity^64^. Higher immune dysfunction scores in the low-TMGS group also supported our hypothesis. Besides, although PDAC has a relatively lower TMB level than other solid tumors, the high-TMGS group is significantly correlated with a higher TMB value, which also supports a more favorable ICB response rate in this group. It seems that TMGS is a reliable biomarker to predict the ICB treatment response of PDAC. How about the therapeutic insights of TMGS in other patients who will not benefit from ICB? We identified that the IC50 values of Gemcitabine, Erlotinib, Paclitaxel, and Doxorubicin positively correlate with the TMGS, indicating that patients in the low-TMGS group might have a better response to these agents. Interestingly, the results also indicated that patients in the high-TMGS group might have a favorable response to Metformin. Recently, Wang et al. reported that Metformin could inhibit pancreatic cancer metastasis caused by *SMAD4* deficiency and consequent *HNF4G* upregulation^54^. Meanwhile, they indicated that Metformin treatment could improve clinical outcomes in patients with *SMAD4*-deficient PDAC in a clinical trial^65^. Taken together, we identified that although patients with high-TMGS have decreased OS compared to low-TMGS, they might have remarkable treatment responses to ICB and Metformin. In contrast, patients with low TMGS have longer OS and could gain clinical benefits from traditional chemotherapeutic agents and targeted therapy. These findings indicate that TMGS is a reliable biomarker to predict the prognosis and guide treatment patterns for patients with PDAC.

There are several inevitable limitations in this study: (1) Although scRNA-*seq* and bulk RNA-*seq* data were combined to develop TMGS based on the mRNA expression level of T cell marker genes, the sample size in the scRNA-*seq* data is limited, and only three subtypes of T cells (mainly memory T cells) were identified after cell annotation; (2) TME is shaped by various of cell types and factors, and there is crosstalk between different components. Therefore, the immune cell infiltration and immune function status estimated by algorithms could not fully represent their actual status in the TME. In this context, well-designed prospective studies should be designed to provide a more profound understanding of this field; and (3) Despite the advantages of scRNA-*seq* and bulk RNA-*seq* analysis and the ideal results obtained in this study, further experimental validation both in human tissue and cell lines should be performed in the future. Searching for reliable biomarkers for cancer survival and cancer immunotherapy prediction is still arduous and needs a long way to go. We hope that our findings can provide specific insights into this field.

## Conclusions

In conclusion, we identified and verified a novel biomarker, TMGS, which could effectively predict the OS and treatment response to different therapeutic agents in PDAC by integrating scRNA-*seq* and bulk RNA-*seq* data. Patients with high TMGS have decreased OS but might have remarkable treatment responses to ICB and Metformin. However, patients with low TMGS have longer OS and could gain clinical benefits from traditional chemotherapeutic agents and targeted therapy. These findings indicate that TMGS is a reliable biomarker to predict the prognosis and guide the treatment pattern for patients with PDAC.

## Supplementary Information

Supplementary Information.

## Data Availability

Bulk RNA-*seq* data and relevant clinical data were obtained from the TCGA (https://portal.gdc.cancer.gov/), ICGC (https://dcc.icgc.org/releases/, ICGC-PACA-CA and ICGC-PACA-AU), and GEO (https://www.ncbi.nlm.nih.gov/, GSE71729, GSE21501, GSE57495, GSE62452, and GSE78229) databases. 10 × scRNA-*seq* data were downloaded from GEO (https://www.ncbi.nlm.nih.gov/, GSE212966) database. Besides, transcriptional data and clinical information of adNSCLC and mUC patients were obtained from the GSE135222 (https://www.ncbi.nlm.nih.gov/) and IMvigor210 for ICB survival and response rate analyses, respectively.
