# Supplementary material for: Single-cell and bulk RNA sequencing identifies T cell marker genes score to predict the prognosis of pancreatic ductal adenocarcinoma
Source: Sci Rep. 2023 Mar 6;13:3684. doi: 10.1038/s41598-023-30972-7 (PMC9988929; doi:10.1038/s41598-023-30972-7)
Supplement: Supplementary file 1 — Supplementary Information. [file 41598_2023_30972_MOESM1_ESM.pdf]

**Supplementary Figure S1**

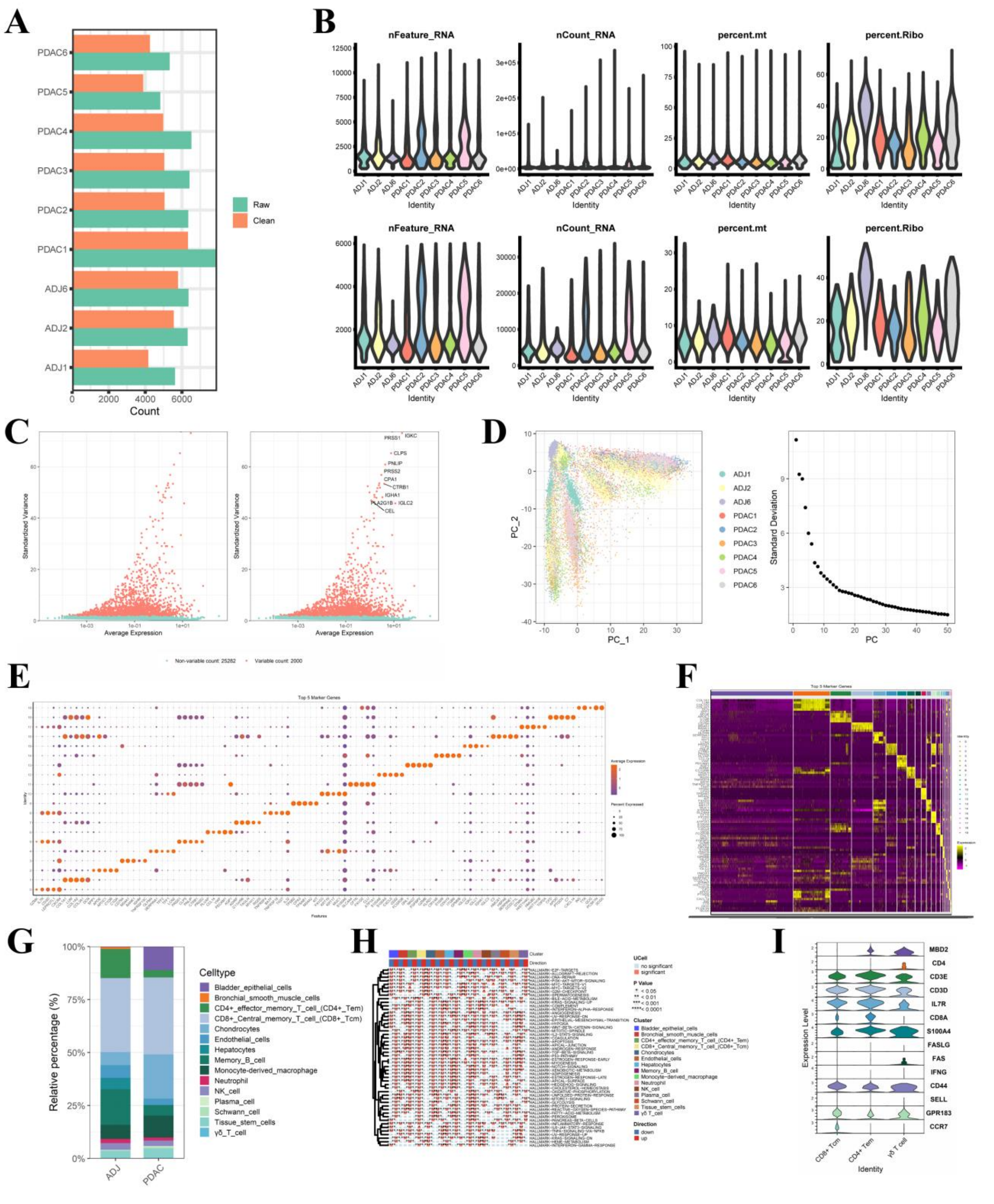


**Supplementary Figure S1. (A-B)** Cell counts and RNA percentages before and after QC. **(C-D)** 2000 highly variable genes identification and PCA. **(E-F)** The dot plot **(E)** and heatmap **(F)** illustrated the top five marker genes of each cluster. **(G)** The proportion of different cell types in PDAC and adjacent samples depicts CD8$^{+}$ Tcm being the most predominant cell type in PDAC and adjacent samples. **(H)**The single-cell GSEA to explore the enriched pathways of each cell type. **(I)** The specific markers genes of CD8$^{+}$ Tcm, CD4$^{+}$ Tem, and γδ T cells. QC, quality control; PCA, principal components analysis; PDAC, pancreatic ductal adenocarcinoma; GSEA, gene set enrichment analysis; Tcm, central memory T cell; Tem, effector memory T cell.

**Supplementary Figure S2**

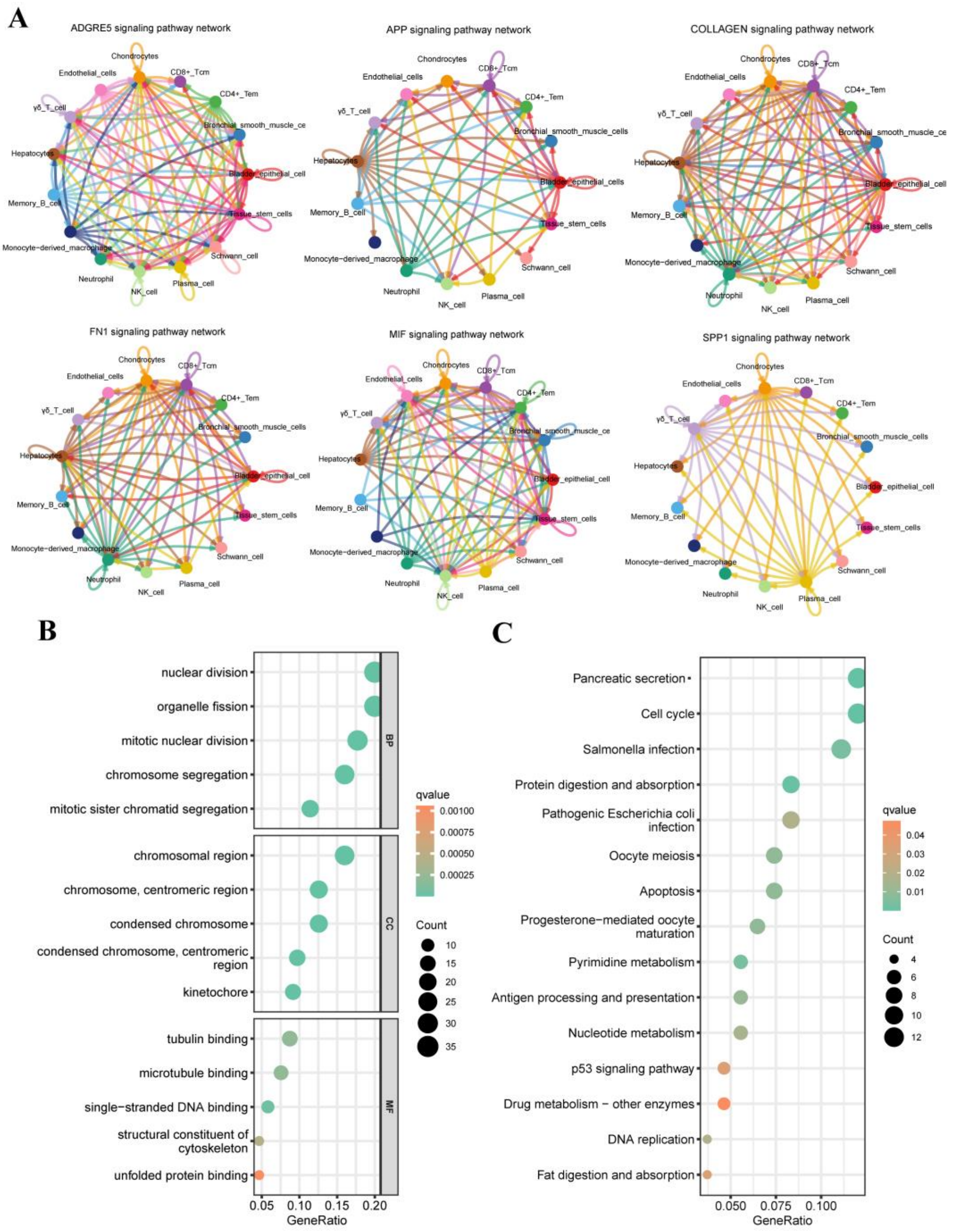


**Supplementary Figure S2. (A)** The cell-cell chat network identified that ADGRE5, APP, COLLAGEN, FN1, MIF, and SPP1 signaling pathways are significantly enriched in the network. **(B)** GO enrichment analysis showed that TMGs were significantly correlated with nuclear division, organelle fission, and mitotic nuclear division. **(C)** KEGG enrichment analysis indicated that TMGs were associated with pancreatic secretion, cell cycle, protein digestion and absorption, apoptosis, antigen processing and presentation, and p53 signaling pathway. GO, Gene Ontology; TMGs, T cell marker genes; KEGG, Kyoto Encyclopedia of Genes and Genomes.

## Supplementary Figure S3

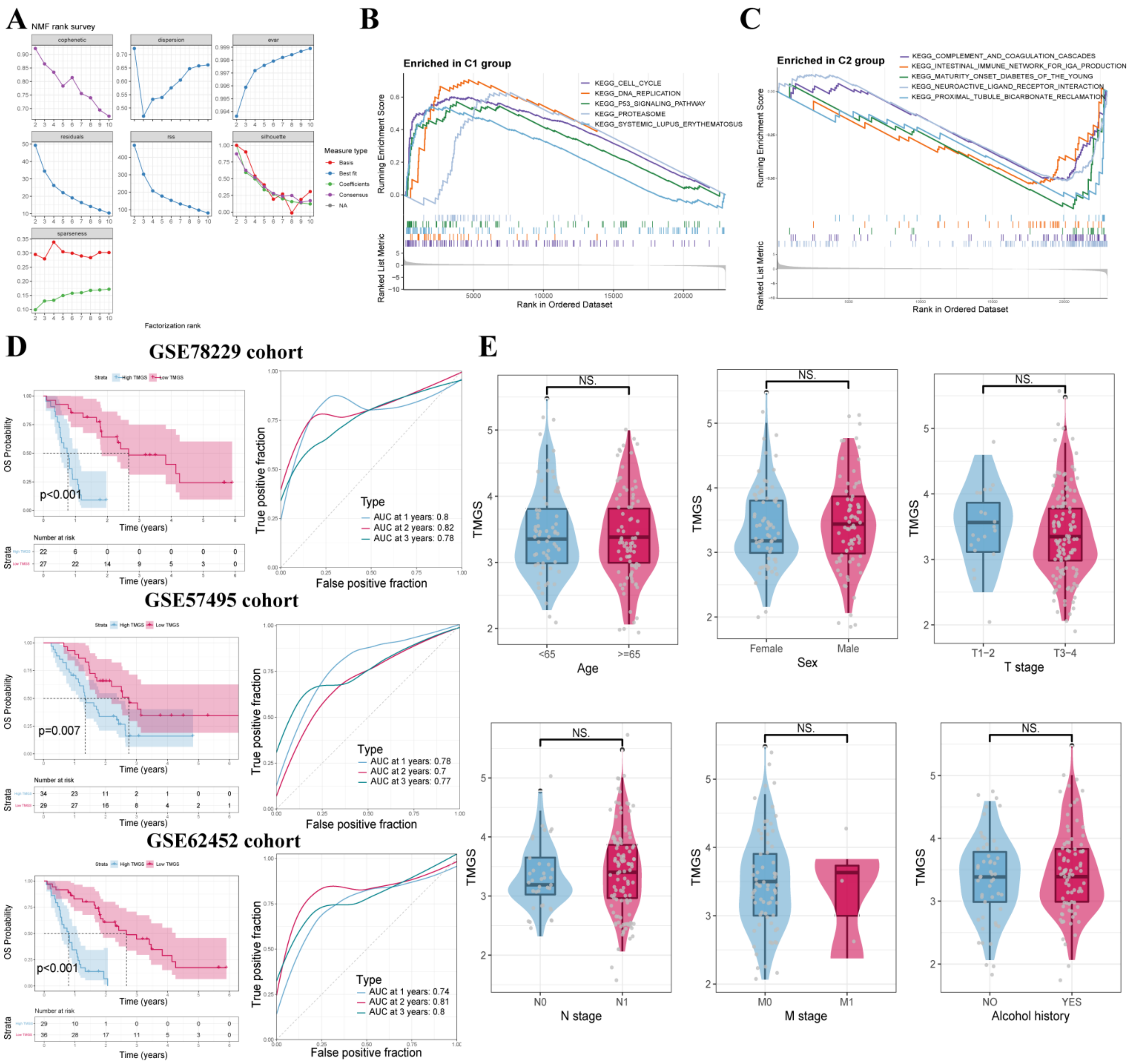


**Supplementary Figure S3. (A)** NMF was performed to decipher heterogeneous molecular clusters based on the TMGs expression matrix in the TCGA cohort. According to parameters such as cophenetic, dispersion, and silhouette, the optimal number of clusters selected was 2. **(B)** GSEA using gene sets from KEGG indicated that C1 was significantly enriched in the cell cycle, DNA replication, and p53 signaling pathways. **(C)** GSEA using gene sets from KEGG indicated that C2 was associated with complement and coagulation cascades and intestinal immune network for IgA production. **(D)** The risk stratification and 1-, 2-, and 3-year OS predictive ability of TMGS in three independent external validation cohorts. (E) There was no statistical difference between TMGS and patients' age, gender, AJCC-T stage, AJCC-N stage, AJCC-M stage, and alcohol history. NMF, non-negative Matrix Factorization; TMGs, T cell marker genes; TCGA; The Cancer Genome Atlas; GSEA, Gene Set Enrichment Analysis; KEGG, Kyoto Encyclopedia of Genes and Genomes; OS, overall survival, TMGS, T cell marker genes score; AJCC, American Joint Committee on Cancer.

## Supplementary Figure S4

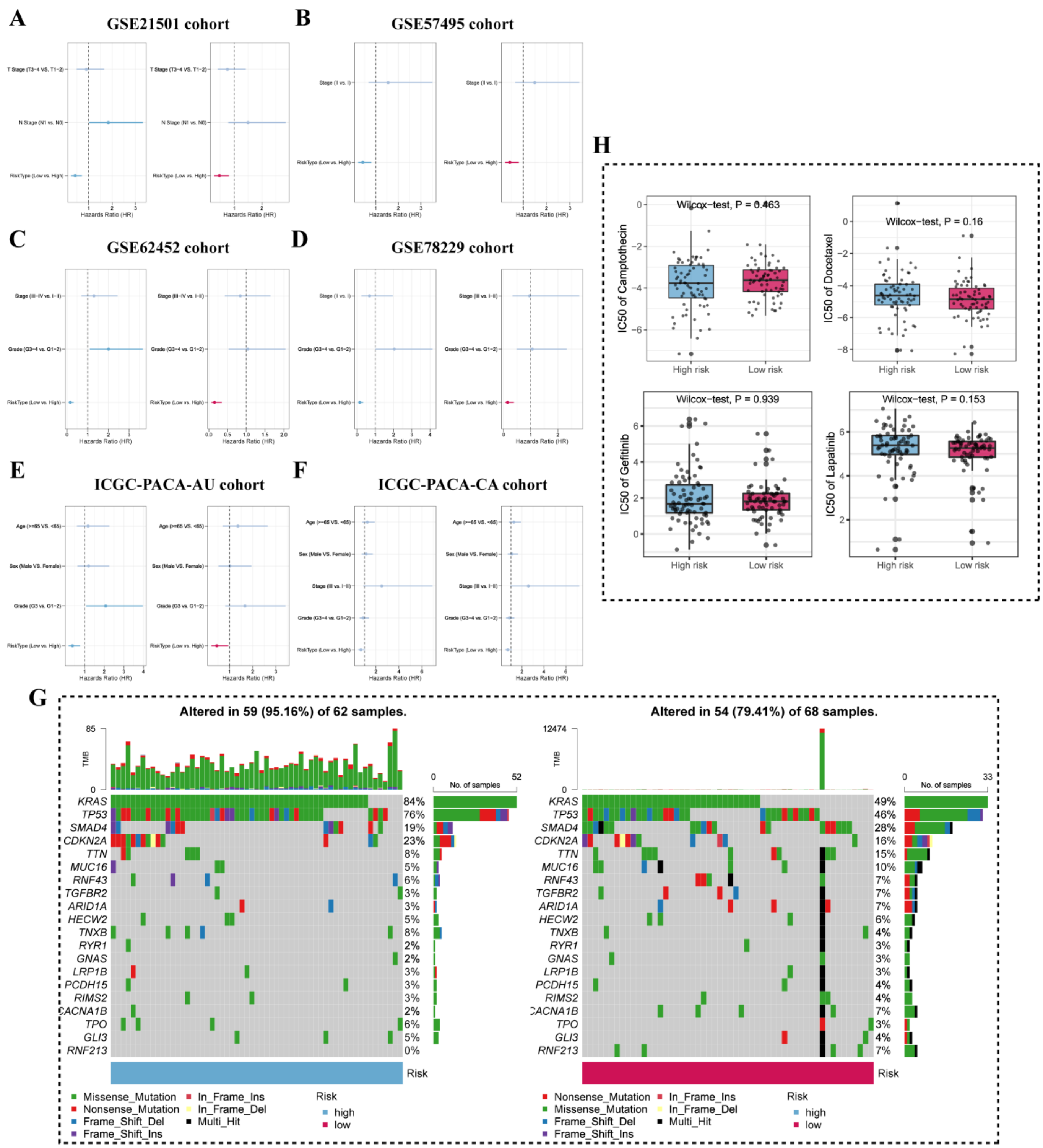


**Supplementary Figure S4. (A-F)** The independent prognostic ability of TMGS in the GSE21501 cohort **(A)**, GSE57495 cohort **(B)**, GSE62452 cohort **(C)**, GSE78229 cohort **(D)**, ICGC-PACA-AU cohort **(E)**, and ICGC-PACA-CA cohort **(F)**. **(G)** The mutation landscape of patients in the high- and low-TMGS groups. (H) The relationship between TMGS and IC50 values of Camptothecin, Docetaxel, Gefitinib, and Lapatinib. TMGS, T cell marker genes score; ICGC, International Cancer Genome Consortium; IC50, semi-inhibitory concentration.

**Supplementary Table S1. The detailed information of each dataset used in this study.**

| **Datasets** | **Sample size** | **Probe ID** | **Purpose** |
|---|---|---|---|
| GSE212966 | | GPL24676 | scRNA-*seq* |
| TCGA-PDAC | 152 | - | Model development |
| ICGC-PACA-CA | 1657 | - | External validation |
| ICGC-PACA-AU | 65 | - | External validation |
| GSE71729 | 125 | GPL20769 | External validation |
| GSE21501 | 97 | GPL4133 | External validation |
| GSE57495 | 63 | GPL15048 | External validation |
| GSE62452 | 65 | GPL6244 | External validation |
| GSE78229 | 49 | GPL6244 | External validation |
| GSE135222 | 27 | GPL16791 | ICB response prediction |
| IMvigor210 | 348 | GPL20301 | ICB response prediction |

Abbreviations: scRNA-*seq*, single-cell RNA sequencing; TCGA; The Cancer Genome Atlas; ICGC, International Cancer Genome Consortium; ICB, immune checkpoint blockade.
